# Flopropione, a Cysteine Conjugate β-Lyase 1 Inhibitor, for Prevention of Cisplatin-Induced Nephrotoxicity: Protocol for a Randomized, Open-Label, Proof-of-Concept Phase 1 and 2a Trial

**DOI:** 10.2196/87907

**Published:** 2026-02-12

**Authors:** Takenao Koseki, Masashi Kondo, Hidetsugu Fujigaki, Kayoko Kikuchi, Yuko Oya, Hiroshi Kato, Tomohiro Mizuno, Naotake Tsuboi, Kenji Kawada, Yasuhiro Goto, Naozumi Hashimoto, Kazuyoshi Imaizumi, Akiko Kada, Hikaru Yabuuchi, Kuniaki Saito, Hideyuki Saya

**Affiliations:** 1 Department of Pharmacy Fujita Health University Hospital Toyoake, Aichi Japan; 2 Department of Regulatory Science for Evaluation and Development of Pharmaceuticals and Devices Graduate School of Medical Sciences Fujita Health University Toyoake, Aichi Japan; 3 Department of Pharmacotherapeutics and Informatics Fujita Health University School of Medicine Toyoake, Aichi Japan; 4 Department of Respiratory Medicine Fujita Health University School of Medicine Toyoake, Aichi Japan; 5 Department of Development and Education of Clinical Research Fujita Health University School of Medicine Toyoake, Aichi Japan; 6 Department of Advanced Diagnostic System Development Graduate School of Medical Sciences Fujita Health University Toyoake, Aichi Japan; 7 Center for Translational Research Fujita Health University Toyoake, Aichi Japan; 8 Department of Nephrology Fujita Health University School of Medicine Toyoake, Aichi Japan; 9 Department of Medical Oncology Fujita Health University School of Medicine Toyoake, Aichi Japan; 10 Center for Society-Academia Collaboration Fujita Health University Toyoake, Aichi Japan; 11 Oncology Innovation Center Fujita Health University Toyoake, Aichi Japan

**Keywords:** cisplatin, cysteine conjugate-β lyase 1, flopropione, kidney injury molecule-1, KIM-1, liver-type fatty acid-binding protein, L-FABP, nephrotoxicity, neutrophil gelatinase-associated lipocalin, N-GAL

## Abstract

**Background:**

Cisplatin-induced nephrotoxicity (CIN) is a major dose-limiting adverse event that can lead to both acute and chronic kidney injury. The formation of thiol-cisplatin conjugates within renal tubular cells has been implicated as a key mechanism underlying CIN. Flopropione is an inhibitor of cysteine conjugate β-lyase 1, an enzyme that catalyzes the formation of the thiol-cisplatin conjugate, which might prevent CIN.

**Objective:**

We designed a clinical trial to evaluate the safety of flopropione in patients receiving cisplatin-based chemotherapy and explore its efficacy in preventing CIN.

**Methods:**

This is a phase 1 and 2a, single-center, randomized, open-label trial conducted in patients undergoing cisplatin therapy. Participants are randomized in a 5:2 ratio per cohort to receive either flopropione or no treatment. On the day of cisplatin administration, the flopropione group receives oral flopropione twice daily (80 mg in cohort 1, 160 mg in cohort 2, and 240 mg in cohort 3). On the following day, all cohorts receive 3 doses of 80 mg of oral flopropione. A step-up dose escalation design is adopted, progressing from cohort 1 to 3 after confirming safety at each level. The primary end point is the safety of flopropione use in combination with cisplatin; the secondary end points include changes in the levels of urinary biomarkers of nephrotoxicity such as neutrophil gelatinase-associated lipocalin, liver-type fatty acid-binding protein, and kidney injury molecule-1. Blood and urine samples are collected within 48 hours before cisplatin administration and at 24 hours, 48 hours, and 1 week after its initiation for safety and efficacy assessments.

**Results:**

The first participant was registered in July 2024. As of January 2026, participant registration is ongoing. The final participant will complete the study by March 2026. Publication of results is expected by March 2027.

**Conclusions:**

This study is expected to contribute to advances in preventive strategies for CIN by providing evidence that inhibition of cysteine conjugate β-lyase 1 by flopropione may attenuate CIN.

**Trial Registration:**

Japan Registry of Clinical Trials jRCTs041220021; https://jrct.mhlw.go.jp/en-latest-detail/jRCTs041220021

**International Registered Report Identifier (IRRID):**

DERR1-10.2196/87907

## Introduction

### Background

Nearly 5 decades have passed since cisplatin was approved in the United States in 1978, and it remains one of the most widely used and effective anticancer drugs worldwide [1]. Cisplatin exerts potent antitumor effects primarily through the formation of DNA cross-links, thereby inhibiting DNA replication and transcription. However, because its cytotoxic activity is not selective for cancer cells, cisplatin damages normal tissues and causes adverse effects, such as myelosuppression, ototoxicity, neurotoxicity, and nephrotoxicity, which often limit its clinical use [2]. Among these toxicities, nephrotoxicity is particularly significant as it can result in both acute and chronic kidney injury [3]. Cisplatin-induced nephrotoxicity (CIN) reportedly occurs in approximately 30% of the patients receiving cisplatin-based chemotherapy [4,5].

Hydration therapy can reduce the intratubular concentration of cisplatin, thereby lowering renal toxicity, and it is recognized as the globally established preventive method for CIN [6,7]. Particularly, recently developed short hydration protocols have offered comparable protective effects to conventional hydration while reducing patient burden and improving safety [8,9]. In addition, magnesium supplementation is used for preventing hypomagnesemia and mitigating the subsequent aggravation of renal dysfunction [10,11]. Use of osmotic diuretics, such as mannitol, has also been reported as a preventive strategy; however, their use might increase risk of nausea, vomiting, and potential deterioration in renal function, particularly in patients with comorbid diabetes mellitus or hypertension [12]. Although emerging preclinical evidence in animal and in vitro studies suggests that antioxidant-based agents, including riociguat, JP4-039, and amentoflavone, exert renoprotective effects against CIN, robust clinical data supporting their efficacy and safety are still lacking [13-15]. Thus, in addition to preventive strategies such as hydration therapy and magnesium supplementation, the development of novel prophylactic approaches for CIN is warranted.

One proposed mechanism underlying CIN involves the formation of a highly reactive thiol-cisplatin complex catalyzed by cysteine conjugate-β lyase 1 (CCBL1) in renal tubular cells [16,17]. We previously reported that 2′,4′,6′-trihydroxyacetophenone, a CCBL1 inhibitor, dose dependently suppressed cisplatin-induced increases in blood urea nitrogen (BUN) and serum creatinine levels and tubular cell damage and apoptosis in a CIN mouse model [18]. Flopropione (2′,4′,6′-trihydroxypropiofenone), identified concurrently with 2′,4′,6′-trihydroxyacetophenone as a CCBL1 inhibitor, exhibits comparable inhibitory activity in renal tubular cells and animal models (Koseki, T, unpublished data, April 2022). Flopropione was approved in Japan in 1978 for its antispasmodic effects in hepatobiliary and pancreatic diseases and urinary calculi [19]. Given its known safety profile and potential to inhibit CCBL1, flopropione may also confer protective effects against CIN.

Therefore, for the first time, a phase 1 and 2a clinical trial has been designed for patients initiating cisplatin therapy to obtain proof of concept for the efficacy and safety of flopropione in preventing CIN.

### Objective

This phase 1 and 2a clinical trial aims to primarily evaluate the safety of flopropione in patients receiving cisplatin-based chemotherapy while exploring preliminary efficacy and generating proof-of-concept evidence for its potential protective effect against CIN.

## Methods

### Study Design and Setting

This is a single-center, randomized, open-label trial conducted at Fujita Health University Hospital (Toyoake, Japan) in patients undergoing cisplatin therapy.

### Eligibility Criteria

Patients who meet all inclusion criteria and none of the exclusion criteria are eligible for enrollment (Textbox 1).

**Inclusion and exclusion criteria.**

**Inclusion criteria**

- Provision of written informed consent before participation after receiving an adequate explanation
- Aged 18 years or older at the time of consent acquisition
- No previous exposure to cisplatin-containing chemotherapy
- Scheduled to receive cisplatin monotherapy or cisplatin in combination with up to 2 other anticancer agents (concurrent radiotherapy permitted) under inpatient care
- Diagnosed with lung cancer, head and neck cancer, or other malignancies scheduled to receive a cisplatin dose of 75 mg/m^2^ or higher

**Exclusion criteria**

- Renal impairment (estimated glomerular filtration rate <60 mL/min/1.73 m^2^)
- Pregnancy or possibility of pregnancy
- Breastfeeding
- Known hypersensitivity to flopropione
- Any other condition deemed inappropriate for study participation by the principal investigator or subinvestigator

No predefined criteria are established for using preventive measures against CIN, including hydration, magnesium supplementation, or mannitol administration. However, at the participating institution, hydration is routinely implemented for managing patients undergoing cisplatin regimens at doses of 75 mg/m^2^ or higher. In addition, magnesium supplementation and mannitol administration are implemented, involving many of the applicable regimens.

### Intervention

#### Intervention Procedure

**Figure 1:**
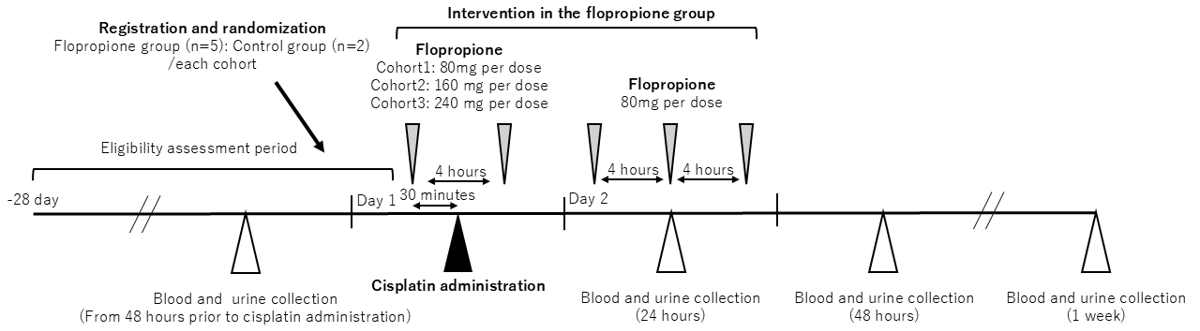
Overview of the study procedure.

**Figure 2:**
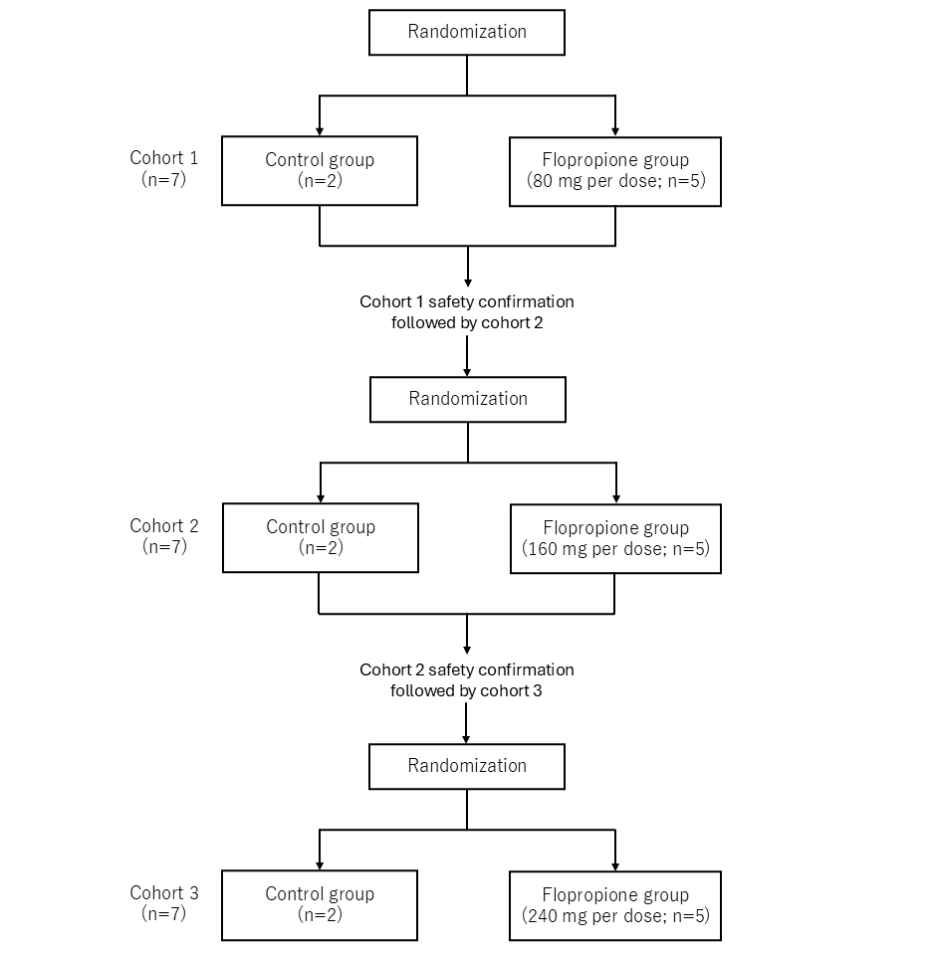
Step-up dosing approach diagram.

Eligible participants are randomized in a 5:2 ratio per cohort to receive either flopropione (flopropione group) or no treatment (control group). Participants in the flopropione group receive oral flopropione twice on day 1 (30 minutes before cisplatin administration and at least 4 hours after) and 3 times on day 2. To ensure participant safety, a step-up dosing approach is applied. The single-dose levels are 80 mg in cohort 1, 160 mg in cohort 2, and 240 mg in cohort 3 on day 1. Progression to the next cohort will occur only after confirming safety in the preceding cohort. On day 2, all participants in the flopropione group will receive 80 mg per dose 3 times daily at intervals of at least 4 hours. Blood and urine samples will be collected within 48 hours before cisplatin initiation and at 24 hours, 48 hours, and 1 week after initiation. Participants in the control group undergo the same procedures as those in the flopropione group and receive standard cisplatin-based chemotherapy but without receiving flopropione. Regarding the analysis, participants assigned to the control group among all cohorts will be pooled for evaluation, whereas participants in the flopropione group will be evaluated according to their assigned dose level. An overview of the study procedure is shown in Figure 1; the step-up dosing approach diagram is shown in Figure 2.

#### Criteria for Discontinuation of the Protocol Treatment

Treatment will be discontinued if any of the following occur: (1) withdrawal of consent by the participant, (2) inability to continue follow-up, and (3) investigator’s judgment that discontinuation is necessary for safety or other reasons.

#### Prohibited Concomitant Drugs

The concomitant use of nonstudy flopropione is prohibited from 1 day before cisplatin initiation until the 1-week postadministration evaluation.

### End Points

#### Primary End Point

Because this is the first clinical use of flopropione in patients treated with cisplatin, the primary end point is safety. Safety will be assessed by the incidence of adverse events, including abnormal laboratory test results, occurring from study treatment initiation (or 30 minutes before cisplatin initiation in the control group) until 1 week after cisplatin administration. Adverse events will be graded according to the Common Terminology Criteria for Adverse Events (CTCAE; version 4.0), while serious adverse events will be defined based on predefined seriousness criteria.

#### Secondary End Points

Secondary end points include changes in urinary biomarkers of nephrotoxicity—neutrophil gelatinase-associated lipocalin (N-GAL), liver-type fatty acid-binding protein (L-FABP), kidney injury molecule-1 (KIM-1), *N*-acetyl-β-d-glucosaminidase (NAG), β2-microglobulin, and α1-microglobulin—from baseline to 24 hours, 48 hours, and 1 week after cisplatin administration.

#### Exploratory End Points

Exploratory end points include the following:

- Changes in serum creatinine, BUN, and estimated glomerular filtration rate from baseline to 24 hours, 48 hours, and 1 week after cisplatin initiation
- Proportion of participants meeting the CTCAE grade 1 or higher acute kidney injury criteria (increase in serum creatinine of >0.3 mg/dL or 1.5 times above baseline) at 24 hours, 48 hours, and 1 week
- Nontargeted metabolomic analyses of serum and urine samples at each time point

### Study Schedule

The schedule for informed consent, registration, randomization, observations, and assessments is presented in Table 1.

**Table 1:** Study schedule.

Items	Before cisplatin administration		After cisplatin administration		
	From 28 d before cisplatin administration (before visit)	Cisplatin administration day (visit 1)	24 h later (visit 2)	48 h later (visit 3)	1 wk later (visit 4)
Allowable visit windows	—^a^	—	–2 h to +2 h	–2 h to +2 h	–36 h to +36 h
Consent acquisition and registration	✓^b^	✓^b^			
Patient background	✓^b^	✓^b^			
Cisplatin administration		✓			
Flopropione treatment		✓^c^	✓^c^		
Blood and urine collection	✓^b,d^	✓^b,d^	✓	✓	✓
Regimen name and concomitant therapy	✓^e^	✓^e^	✓^e^	✓^e^	✓^e^
Adverse events		✓^f^	✓^f^	✓^f^	✓^f^

^a^Not applicable.

^b^Conduct at either the before visit or visit 1.

^c^The first dose is taken 30 minutes before starting cisplatin administration. On the day of administration, a second dose is taken at least 4 hours apart. On the second day, 3 doses of 80 mg are taken at least 4 hours apart.

^d^Data are collected from 48 hours before cisplatin administration until before starting the regimen.

^e^Data collection period is from registration to 1 week after cisplatin administration.

^f^Data are collected from the start of flopropione treatment (or 30 minutes before starting cisplatin administration in the control group) until the 1-week evaluation point or until discontinuation. As much data as possible are collected at the discontinuation point. If adverse events persist, observation is continued until recovery or until the principal investigator or equivalent determines follow-up is unnecessary.

### Eligibility Assessment

Eligibility will be confirmed within 28 days before cisplatin initiation. Baseline characteristics, including age, sex, height, weight, and comorbidities, will be recorded at registration.

### Registration and Random Allocation

After confirming eligibility, registration and randomization will be performed through an electronic data capture system, assigning participants in a 5:2 ratio (flopropione:control) within each cohort.

### Flopropione Treatment

Flopropione (80 mg per tablet) will be administered orally 30 minutes before cisplatin initiation. Doses are as follows: 80 mg in cohort 1, 160 mg in cohort 2, and 240 mg in cohort 3. A second dose of the same amount will be given at least 4 hours later on day 1, followed by 80 mg 3 times daily on day 2 at intervals of at least 4 hours.

### Blood and Urine Collection

Blood and urine samples will be collected at the time points defined as the secondary and exploratory end points. The details of each test item are summarized in Table 2.

**Table 2:** Laboratory test items.

Classification	Items
Hematological tests	White blood cell count, neutrophil count, lymphocyte count, monocyte count, eosinophil count, basophil count, red blood cell count, hemoglobin, hematocrit, and platelet count
Blood biochemical tests	Total cholesterol, triglycerides, total protein, albumin, albumin/globulin ratio, blood urea nitrogen, uric acid, creatinine, total bilirubin, aspartate aminotransferase, alanine aminotransferase, γ-glutamyl transpeptidase, alkaline phosphatase, lactate dehydrogenase, creatinine kinase (creatine phosphokinase), calcium, phosphorus, sodium, potassium, chlorine, and C-reactive protein
Urinalysis	Hematuria, glucose, protein, creatinine, neutrophil gelatinase-associated lipocalin, liver-type fatty acid-binding protein, kidney injury molecule-1, *N*-acetyl-β-d-glucosaminidase, β2-microglobulin, and α1-microglobulin

### Biomarker Measurements

The collected urine samples will be centrifuged at 400×*g* or higher for 5 minutes. Thereafter, the supernatants will be stored at –80 °C until analysis. The samples will be sent to LSI Medience Corporation for N-GAL, L-FABP, KIM-1, NAG, β2-microglobulin, and α1-microglobulin measurement. N-GAL will be measured by a chemiluminescent immunoassay; L-FABP, β2-microglobulin, and α1-microglobulin will be measured by latex agglutination turbidimetry; KIM-1 will be measured by an enzyme-linked immunosorbent assay; NAG will be measured by the artificial substrate MPT method.

### Adverse Events

All adverse events, including abnormal laboratory findings, will be collected and recorded in the electronic medical records with information on severity, seriousness, outcome, and the presence or absence of a causal relationship. The seriousness criteria for serious adverse events are (1) death, (2) life-threatening complication, (3) patient requires inpatient hospitalization or prolongation of hospitalization, (4) persistent or significant disability or incapacity, (5) risk of persistent or significant disability or incapacity, (6) congenital anomaly or birth defect, (7) and important medical event.

### Sample Size

The planned sample size includes 21 participants: 5 (24%) each in the 80 mg, 160 mg, and 240 mg flopropione cohorts and 6 (29%) in the control group. On the basis of the number of eligible patients in the previous year, anticipated consent rate, and screening dropouts, 21 participants are considered feasible to complete enrollment within 1 to 1.5 years.

Given the early-phase pilot nature of this study, the sample size is selected to balance between exploratory evaluation of potential dose-response trends, safety assessment, ethical considerations, and feasibility. Although not powered for statistical significance, 5 participants per dose level is considered appropriate for an initial exploration of a proof-of-concept study. Participants assigned to the control group will be enrolled among all cohorts and pooled for assessment, providing a common reference group for comparisons with each dose level. This approach allows for efficient use of control data while minimizing unnecessary patient exposure to the investigational intervention.

### Statistics

#### Analysis Set

##### Full Analysis Set

Patients in the control group are those who receive cisplatin, and participants in the flopropione group are those who receive flopropione. Data of participants in the flopropione group who do not receive flopropione are not included in the analysis.

##### Per Protocol Set

This is the subset of the full analysis set that excludes participants who have deviations in drug administration or those who use prohibited concomitant medications.

##### Safety Analysis Set

This set is the same as the full analysis set. The control group is evaluated as 1 group, and the flopropione group is stratified by dose.

#### Analysis Method

As this is an exploratory pilot study, no formal hypothesis testing is planned. Descriptive and exploratory analyses will be conducted as mentioned subsequently.

The changes in urinary N-GAL, L-FABP, KIM-1, NAG, β2-microglobulin, and α1-microglobulin as well as serum creatinine, BUN, and estimated glomerular filtration rate from baseline to 24 hours, 48 hours, and 1 week after cisplatin administration will be assessed. Summary statistics will be calculated for the change amount, change rate, and observed values at each time point (24 hours, 48 hours, and 1 week after administration) relative to the baseline for each marker. Additionally, between-group differences will be estimated using mixed-effects models for repeated measures if feasible, following an assessment of the data distribution before database lock.

The proportion of participants meeting the CTCAE grade 1 or higher acute kidney injury criteria (creatinine level increase of 0.3 mg/dL or higher; creatinine 1.5 times above baseline) at 24 hours, 48 hours, and 1 week after cisplatin administration will be calculated. The number of participants meeting this criterion at each time point will be analyzed using Fisher exact test.

Nontargeted metabolome analysis will be conducted on serum and urine samples collected at each time point, following previously described protocols [20,21]. Briefly, metabolites will be extracted from the samples and analyzed using capillary electrophoresis coupled with time-of-flight mass spectrometry (Agilent 6210, Agilent Technologies). Metabolite identification will be performed by matching accurate mass-to-charge ratios (m/z) and migration times to the reference annotation table provided by Human Metabolome Technologies Inc. Peak areas obtained from the capillary electrophoresis coupled with time-of-flight mass spectrometry analysis will be normalized and expressed as relative concentrations to enable comparative evaluation across samples. Statistical analyses will include both univariable approaches and multivariable methods to identify significant temporal changes in the metabolome.

Differences in cancer type, cisplatin dose, renal function, and comorbidities may potentially influence the study end points. Therefore, to explore the impact of these baseline characteristics and clinical variables, subgroup analyses may be considered when deemed necessary.

### Data Management

The data will be collected using an electronic data capture system. All data and materials obtained in this study will be managed under the responsibility of the principal investigator. Digital data will be encrypted with passwords, and paper-based materials will be securely stored in locked cabinets. Data will be retained for 5 years following the completion of this clinical study. Upon disposal, all materials will be destroyed in an irreversible manner through methods such as shredding or incineration to ensure destruction.

### Ethical Considerations

This study will be conducted in accordance with Japan’s Clinical Trials Act and the Declaration of Helsinki. The protocol was reviewed and approved by the clinical research review board of Fujita Health University (CR22-002). At Fujita Health University Hospital, the principal investigator or subinvestigator will screen patients under their care to identify those who may meet the eligibility criteria for participation in this study. Written informed consent will be obtained from eligible patients by the principal investigator or subinvestigator using an explanatory document approved by the clinical research review board, within 28 days before cisplatin administration. All participant data will be deidentified and maintained confidentially. Data will be stored securely with restricted access. Data will be retained for 5 years after study completion. For adverse events, the principal investigator or subinvestigator will provide appropriate medical treatment. For severe adverse events, clinical research insurance will provide appropriate compensation to participants.

### Dissemination

The results will be disseminated through peer-reviewed publications, conference presentations, and the Japan Registry of Clinical Trials (jRCTs041220021).

### Monitoring and Auditing

On-site monitoring will be performed to ensure compliance with the study protocol, participant safety, and data accuracy. No formal audit is planned.

### Trial Registration

This trial was registered in the Japan Registry of Clinical Trials (jRCTs041220021).

## Results

This study was approved by the clinical research review board of Fujita Health University in April 2022. This study was subsequently funded by the Japan Agency for Medical Research and Development in April 2024, with the first case registered in July 2024. As of January 2026, participant registration is ongoing. The final participant will complete the study by March 2026. Publication of results is expected by March 2027.

## Discussion

### Anticipated Findings

CIN is a critical concern about initiating or continuing cisplatin-based chemotherapy. Although hydration therapy is widely implemented, its preventive effects against CIN remain limited. Various pharmacological agents have been investigated for potential protective effects; however, none have yet been successfully translated into clinical practice, and an unmet medical need persists [22]. In addition to currently recommended preventive strategies, such as hydration, the development of mechanism-based pharmacological interventions targeting the pathogenesis of CIN not only reduces the incidence and severity of CIN but also enables cisplatin therapy continuation, potentially leading to improved overall treatment outcomes. Therefore, we designed a clinical trial to evaluate the safety of flopropione, a CCBL1 inhibitor, and obtain proof of concept for its preventive effect against CIN. If proof of concept for the preventive effect of flopropione can be demonstrated in this study, subsequent clinical trials would be able to further verify its efficacy. Such evidence would support the potential of flopropione as an adjunctive therapy to existing preventive strategies, including hydration, with the expectation of achieving greater suppression of CIN incidence compared with those currently available. Furthermore, if its clinical benefit is confirmed, future studies should also consider comparative trials against hydration-based protocols to evaluate whether flopropione could serve as a simpler and more convenient alternative preventive approach to CIN.

In designing this study, several measures are implemented to ensure participant safety while obtaining proof of concept. No previous studies have evaluated the concomitant use of flopropione with cisplatin; therefore, a step-up dosing approach was adopted in the participants who were hospitalized. This design allows for sequential confirmation of safety across cohorts while gradually increasing the flopropione dose. Given that hydration may reduce plasma concentrations of orally administered drugs, we have set the maximum single dose of flopropione on cisplatin administration day 1 at 240 mg, consistent with the previously reported dose administered to healthy adults [19].

N-GAL, L-FABP, and KIM-1 were selected as urinary biomarkers for detecting early renal injury. All have been reported as useful indicators for early detection of CIN [23-25]. Furthermore, urinary KIM-1 levels reportedly continue to rise for up to 10 days after the initial administration of cisplatin [26]. Therefore, urinary biomarkers will be measured at 24 hours, 48 hours, and 1 week after cisplatin initiation.

### Limitations

This study has several limitations. First, it is an open-label study using a nontreated control group rather than a placebo control. Although the primary focus is on safety and biomarker evaluation, the potential for participant- or investigator-related bias cannot be completely eliminated. Therefore, a future pivotal study should adopt a randomized, double-blind, placebo-controlled design. Second, this study does not assess the impact of flopropione on cisplatin efficacy. Because this is the first clinical investigation of flopropione used in combination with cisplatin, it is limited to the first cisplatin administration (cycle 1) with an observation period of 1 week. Evaluation of potential effects on antitumor efficacy will require a longer observation period. Third, several studies have suggested that nephrotoxicity develops in a cumulative, dose-dependent manner [27,28]. Therefore, CIN may not necessarily occur after a single cisplatin administration. Accordingly, future studies involving multiple cycles of cisplatin and repeated treatment of flopropione will be necessary for comprehensively assessing its safety and preventive efficacy.

### Conclusions

Cisplatin continues to be one of the most important anticancer agents. However, prevention of CIN is crucial for expanding therapeutic options in patients with cancer and enhancing treatment sustainability. This study is a proof-of-concept trial designed to evaluate flopropione, a CCBL1 inhibitor, with a focus on the pathophysiological mechanisms underlying CIN. It occupies an important position in generating evidence that will inform subsequent clinical trials. Through this study and the following clinical trials, we aim to demonstrate the preventive efficacy of flopropione against CIN and establish it as a prophylactic agent capable of further reducing the incidence of CIN.

## Data Availability

The datasets generated or analyzed during this study are not publicly available due to participant confidentiality and ongoing regulatory requirements, but are available from the corresponding author on reasonable request, subject to approval by the institutional review board.

## Abbreviations

BUN: blood urea nitrogen
CCBL1: cysteine conjugate-β lyase 1
CIN: cisplatin-induced nephrotoxicity
CTCAE: Common Terminology Criteria for Adverse Events
KIM-1: kidney injury molecule-1
L-FABP: liver-type fatty acid-binding protein
NAG: N-acetyl-β-d-glucosaminidase
N-GAL: neutrophil gelatinase-associated lipocalin
